# Familiar but neglected: identification of gaps and recommendations to close them on exclusive breastfeeding support in health facilities in Malawi

**DOI:** 10.1186/s13006-021-00418-9

**Published:** 2021-09-26

**Authors:** Alinane Linda Nyondo-Mipando, Mai-Lei Woo Kinshella, Sangwani Salimu, Brandina Chiwaya, Felix Chikoti, Lusungu Chirambo, Ephrida Mwaungulu, Mwai Banda, Laura Newberry, Tamanda Hiwa, Marianne Vidler, Queen Dube, Elizabeth Molyneux, Joseph Mfutso-Bengo, David M. Goldfarb, Kondwani Kawaza

**Affiliations:** 1 School of Public Health and Family Medicine, Department of Health Systems and Policy, College of Medicine, University of Malawi, Blantyre, Malawi; 2 Department of Obstetrics and Gynaecology, BC Children’s and Women’s Hospital and University of British Columbia, Vancouver, Canada; 3 Department of Pediatrics and Child Health, College of Medicine, University of Malawi, Blantyre, Malawi; 4 Queen Elizabeth Central Hospital, Pediatrics, Blantyre, Malawi; 5 Center of Bioethics for Eastern & Southern Africa (CEBESA), Blantyre, Malawi; 6 Department of Pathology and Laboratory Medicine, BC Children’s and Women’s Hospital and University of British Columbia, Vancouver, Canada

## Abstract

**Background:**

Exclusive breastfeeding is widely accepted as a key intervention with proven efficacy for improving newborn survival. Despite international commitments and targets to support and promote breastfeeding, there are still gaps in meeting and maintain coverage in many sub-Saharan African countries. This paper aimed to triangulate the perspectives of health workers, mothers, and their family members with facility assessments to identify gaps to improve breastfeeding support in in Malawi.

**Methods:**

The study on breastfeeding barriers and facilitators was conducted in 2019 at one tertiary hospital and three secondary-level hospitals in Malawi. We conducted 61 semi-structured interviews with health workers, postnatal mothers, grandmothers, aunts, and fathers. In 2017, we carried out a neonatal care facility assessment using the World Health Organization (WHO) Integrated Maternal, Neonatal, and Child Quality of Care Assessment and Improvement Tool. Qualitative data were analysed using a thematic analysis approach within the Systems Framework for Health Policy.

**Results:**

The district-level hospitals rated high with an average score of 4.8 out of 5 across the three facilities indicating that only minor improvements are needed to meet standards of care for early and exclusive breastfeeding. However, the score fell to an average of 3.5 out of 5 for feeding needs with sick neonates indicating that several improvements are needed in this area. The qualitative data demonstrated that breastfeeding was normalized as part of routine newborn care. However, the focus on routine practice and reliance on breastfeeding knowledge from prenatal counselling highlights inequities and neglect in specialized care and counselling among vulnerable mothers and newborns. Revitalisation of breastfeeding in Malawian facilities will require a systems approach that reinforces policies and guidelines; contextualises knowledge; engagement and empowerment of other relatives to the baby and task-sharing among health workers.

**Conclusions:**

Breastfeeding is accepted as a social norm among health workers, mothers, grandmothers, aunts, and fathers in Malawi, yet vulnerable groups are underserved. Neglect in breastfeeding support among vulnerable populations exacerbates health inequities. Health systems strengthening related to breastfeeding requires a concerted effort among health workers, mothers, grandmothers, aunts, and fathers while remaining grounded in contexts to support family-centered hospital care.

## Background

Exclusive breastfeeding is widely accepted as a key intervention with proven efficacy for improving newborn survival [1, 2]. Properly implemented breastfeeding is estimated to reduce all-cause neonatal mortality and morbidity by 55–87% [1], and scaling up breastfeeding to near-universal levels (90% coverage) is estimated to save 823,000 deaths of children under the age of two (13.8% of all deaths) per year in high mortality low- and middle- income countries (LMICs) [3]. In addition to the recognized short-term benefits, breastfeeding also has long-term benefits on human capital including improved intelligence, which may impact educational attainment and income in adulthood [4]. The Innocenti Declaration in August 1990 affirmed recommendations for exclusive and continued breastfeeding, the Baby-Friendly Hospital Initiative (BFHI) was launched in 1991, and support of breastfeeding was reaffirmed as an essential part of achieving the Millennium and Sustainable Development Goals. However, despite international commitments and targets to support and promote breastfeeding, global breastfeeding rates remain far below international targets [5]. A 2018 study found that only 18 out of 49 African countries were on track to meet the WHO Global Nutrition Targets 2025, to increase the rate of exclusive breastfeeding in the first six months of life to 50 % [6]. A further analysis that categorised each country according to its progress towards improving in exclusive breastfeeding rates reported that Malawi needs to improve from 59 to 69% by 2025 [7]. Despite the availability of impressive policies and documents that support breastfeeding, there has been suboptimal reinforcement by healthcare workers [8, 9].

Several factors have been suggested for improving implementation of breastfeeding and these include training of staff [10, 11] with emphasis on skills and confidence for health workers to support women [8], comprehensive maternal counseling, periodic assessment of the facilities to assess compliance [11] and supportive supervision [10, 11].

This paper aimed to triangulate the perspectives of health workers mothers, grandmothers, aunts, and fathers with facility assessments to identify gaps that can be addressed to improve breastfeeding support in Malawi.

The information will inform policymakers revitalizing breastfeeding in Malawi because it highlights the areas that need strengthening.

## Methods

### Study design

This is a convergent mixed-method study drawing from a cross-sectional quantitative study and a descriptive qualitative study [12]. This design entails employing both quantitative and qualitative data approaches in collecting the data and keeping the data sets separate and mixing them at the point of interpretation to highlight the points where the two data sets converge or diverge [12]. Specifically in this study, the quantitative component employed a facility assessment on the status of the routine care environment and capacities for neonatal care in the study sites while the qualitative component employed interviews with health workers, postnatal mothers, grandmothers, aunts and fathers. The study was done under a larger project titled “Integrating a neonatal healthcare package for Malawi”, funded as part of the Innovating for Maternal and Child Health in Africa (IMCHA) initiative by the Canadian International Development Research Centre (IDRC), Global Affairs Canada (GAC) and the Canadian Institutes for Health Research (CIHR). Ethics approvals were obtained from the University of Malawi College of Medicine (P.08/15/1783) and the University of British Columbia (H15–01463-A003).

### Research setting

The qualitative component of the study was conducted at one tertiary and three secondary-level hospitals in southern Malawi and these have been described earlier [13]. The facility assessment was conducted in the same three secondary-level hospitals, which included two government facilities and a mission hospital owned by the Christian Health Association of Malawi [14]. We excluded the tertiary facility in the facility assessment because most deliveries occur in district facilities necessitating the need for this review in those settings [13].

### Recruitment of study participants

Participants were recruited following pre-determined criteria to include a purposive sample of health professionals engaged in service delivery or supervision of clinical newborn care at the facilities as well as mothers, fathers, and other family members of breastfeeding at the facility. We included mothers of infants deemed stable by the head nurse in the ward and employed a snowball approach to reach out to fathers and other family members such as grandmothers through breastfeeding mothers.

Three potential participants refused to participate, with one mother citing unpreparedness as a reason for non-willingness, a grandmother who felt she did not have anything to share regarding the subject in question, and a male partner who cited time challenges. The three were thanked for their time and assured that their decision would not affect the care of their babies.

### Data collection and management

Prior to any data collection procedures, all researchers introduced themselves as members of IMCHA, a research group at the College of Medicine University of Medicine (CoM). The facility assessment methodology is described in our earlier paper [14] but briefly this was conducted using an adaptation of the WHO Integrated Maternal, Neonatal, and Child Quality of Care Assessment and Improvement Tool. The facility assessment followed the four steps outlined by the tool and included a meeting with the hospital management and clinical staff, an assessment done by walking around the facility, observations following the guide, and then meetings to discuss preliminary findings to outline gaps and progress [14]. The assessment was done in November 2017.

The qualitative data were collected between April and June 2019 through face-to-face semi-structured interviews of 30–60 min at each of the four health facilities. We obtained informed consent from each participant before each interview. Data were collected by three certified nurse-midwife-technicians (authors BC, FC, and EM) and two public health specialists (authors SS and LC). Four of the researchers were female and one was male and they all had no prior relationship or encounter with the study participants. Interviews were audio-recorded with permission in the language of preference for participants (mainly Chichewa for postnatal mothers, grandmothers, aunts and fathers and a mixture of English and Chichewa for health workers).

All data were stored according to the University of Malawi College of Medicine’s regulations in locked cabinets and password-protected computers.

### Quantitative data analysis

Facility assessment data were initially captured on paper at the point of data collection, which were scanned and transferred to a REDCap database (Vanderbilt University, Nashville, USA). We conducted data quality control by two independent researchers double entering the data and comparing to assess inconsistencies. As part of the tool, summary scores for each section were calculated as an average of responses on a five-point Likert scale using Excel (Microsoft Corporation, Redmond, USA). A score of one indicated services not provided or dangerously inadequate care while scores of four or five were considered acceptable, as little improvements needed or meet standards of care, respectively [14].

### Qualitative data analysis

Interviews were transcribed in verbatim with simultaneous translation to English where needed. Transcripts underwent a two-step process of quality control where first, they were checked against the audio by the qualitative research coordinator (SS), and secondly, a random sample was checked against the audio by ALMN. Qualitative data were managed using NVivo12 (QSR International, Melbourne, Australia). We identified all our participants using codes.

Qualitative data were analysed using a thematic approach [15] within the Systems Framework for Health Policy, which includes interrelated conceptual blocks of Governance, Advocacy, Capacity, and Knowledge [16]. Governance consists of legislation in terms of policies, funding, and organisation of services, quality assurance, transparency, and auditing. Knowledge aims at gathering and analysing current information to understand the determinants of health. Advocacy is aimed at promoting the policy and capacity involves the people that are involved in implementing the policy. The coding guide was developed following the system’s framework for health policy and was discussed iteratively between MWK and ALNM. Transcripts were primarily coded by SS with a second round of deductive coding by MWK and ALNM for quality control. Specifically, after the coding process, all data were sorted to ensure that they are apportioned appropriately as per codes within the System’s framework for health policy through an iterative process. Data that were not fitting were either assigned to another code or discarded if they were not well aligned. Once the initial assignment was done, it was reviewed by the research team for consistency and accuracy and elimination of overlapping ideas. All themes were verified and refined to ensure consistency with the theme.

## Results

### Facility assessment

The district-level hospitals had an average score of 4.8 (4.6 to 5) out of 5 across the three facilities for early and exclusive breastfeeding, which indicates largely complying with standards of care with only minor improvements needed (see Table 1). Observations confirmed scores that mothers were counselled by health professionals after delivery to initiate breastfeeding within the first hour and promote exclusive breastfeeding practices. However, while information was provided to mothers as a part of routine care, there may be some gaps in health xworker training to correctly assist breastfeeding initiation and the level of breastfeeding practice remains unclear as no documentation was kept in any of the three district hospitals. The score for feeding needs with sick neonates was an average of 3.5 (3.3 to 3.8) out of 5 across the three facilities, indicating that several improvements are needed to meet standards of care (see Table 1).

**Table 1:** Newborn feeding practices in facility assessment

	District 1 – a government hospital	District 2 – mission hospital	District 3 – a government hospital
The newborn baby is put on the abdomen or to the breast for skin contact immediately after birth if no need for resuscitation	5	5	5
Initiation of breastfeeding is encouraged within the first hour, and mothers are given a quiet atmosphere to do so	5	5	4
Staff are trained to assist mothers and babies in initiating breastfeeding correctly	5	4	4
Expressed breastmilk is given by cup or nasogastric tube when the child is unable to feed or if the mother cannot stay with the child all the time	5	5	3
There is no promotion of infant formula on the ward and samples are not distributed to mothers or staff	5	5	5
Infant formula, glucose supplementation, water supplementation are not used unless upon written medical instruction	5	5	5
Exceptions to exclusive breastfeeding are based on current evidence	5	5	5
There are no restrictions on the frequency or length of breastfeeding	5	5	5
At discharge, exclusive breastfeeding is recommended until the age of 6 months and complementary breastfeeding until 24 months	5	4	5
Documentation on breastfeeding and frequency is kept	1	1	1
**Early and exclusive breastfeeding summary score**	5	4.8	4.6
All preterm and low birthweight are admitted for monitoring (< 2000 g)	3	5	3
Mothers are NOT routinely separated from babies, and can room-in together	5	5	2
Specific feeding needs of low birthweight and other sick newborns are taken care of	3	2	2
There are feeding protocols by day/weight, etc.	5	1	5
Special feeding needs are included in the patient plan/record	5	3	3
Mothers’ milk is given to low birthweight (LBW) babies as much as possible	4	5	4
Frequent feedings (at least 8 per day) are provided to LBW babies and intake is monitored	5	3	3
Babies unable to feed are given expressed breast milk by cup, spoon, or fed by nasogastric tube in adequate amounts according to age. Intake is monitored	4	4	4
Guidelines for recognition and treatment of hypoglycemia are available, known, and used by staff	4	4	4
**Feeding needs for sick neonates summary score**	3.8	3.3	3.5

Observations note that while feeding protocols existed in two of the three facilities and mothers roomed together with their newborns, there were overall gaps in monitoring and regular clinical reassessment by physicians. Specific feeding needs of low birthweight and other sick newborns were poorly considered and intake was rarely recorded in inpatient charting.

### Qualitative findings

#### Participant characteristics of health workers

We interviewed 31 healthcare workers (healthcare workers include Nurses/Midwives, Clinical Offficers who are are technical officers that are trained in clinical medicine at the diploma level and Medical Doctors). Specifically, we included 20 nurses, seven Clinical Officers, and four Medical Doctors. Their age ranged from 25 to 75 years while their work experience ranged from six months to 56 years. Sixteen were trained to offer breastfeeding support. There were 24 interviews from district hospitals and seven from the tertiary hospital.

#### Participant characteristics of postnatal mothers, grandmothers, aunts and fathers

We interviewed 30 postnatal mothers, grandmothers, aunts and fathers and of these 20 were women and 10 were fathers of the admitted baby. Of the 20 women, two were grandmothers and one was an aunt. Their age ranged from 18 to 45 years, all the fathers were either employed or conducting some small scale business while four women were housewives. The duration of stay in the hospital of their baby ranged from three to 21 days. There were 19 interviews from district hospitals and 12 from the tertiary hospital.

#### Perceptions on breastfeeding

There were varied perceptions regarding breastfeeding where on one hand it was regarded as a familiar occurrence and on the other as an aspect that is neglected.

##### Breastfeeding as familiar

Health workers and mothers interviewed in the study shared familiarity with breastfeeding recommendations. Mothers and health workers described breastfeeding as a social norm to practice immediately after delivery, even without postnatal counselling. Some saw it as a natural occurrence without much effort or need for extensive health provider support. Even grandmothers (mothers-in-law and maternal grandmothers) who are the custodians and counsellors in maternity issues in the communities spoke of breastfeeding as a normal occurrence in their communities.

*“I think breastfeeding as an intervention is just an automatic thing, is something which is known by even the mother. She knows exactly that when the baby is born she is supposed to start breastfeeding.” District hospital nursing officer*

*“I didn’t think anything because in Malawi when growing up you see that all the time babies breastfeeding so you always know that if you have a baby you will breastfeed. So when it was my turn, I don’t think I even thought much about it.” District hospital mother*

#### Benefits of breastfeeding

The familiarity of breastfeeding was further supported when participants expressed knowledge on the benefits of breastfeeding and it was reported to be widely accepted among health workers, mothers, and local communities.

*“When a baby is not breastfeeding, they get sick often but breast milk, it’s a protector for the child because it is loaded with all nutrients needed by the baby. That is why exclusive breastfeeding is promoted. We have less and fewer cases of sickness among babies . . . ” District hospital nurse*

*“Community and religious leaders say that exclusive breastfeeding is good as it contains nutrients for the babies to gain weight and grow healthy.” Father at a district hospital*

#### Breastfeeding as neglected

##### Breastfeeding taken for granted

Though familiar, breastfeeding support was also described as taken for granted and neglected. Health professionals at the hospital sometimes assumed that women previously learned about breastfeeding during antenatal care and accept the practice. Lessons on breastfeeding in the postnatal ward may be minimal.

*“I think my point was slight back to attitude, taking things for granted . . . because what is normally focused on is how the mother is . . . We have left the mother who is not bleeding, who is fine, the vitals are fine and is breastfeeding and you just take it for granted that everything is alright but we don’t know this. Is it (breastfeeding) happening? . . . Is it being done the right way? Something like that . . . we take it for granted most of the time.” District hospital nurse*

However, this position by health workers may miss women that need additional support and some mothers are left to learn on their own. A woman who had a baby shared the following experience:

*Interviewer: “So from the day you delivered to date, the medical personnel haven’t still come to tell you about the intervention of lactation support or to assist you?*

*Mother: “No they haven’t.”*

*Interviewer: “You are still using your parents’ ideas on how to do it?”*

*Mother: “Yes I am using the same ideas but most of the time when we see some posters on the walls we also read and follow those instructions.” District hospital, first-time mother*

#### Neglect among special cases

The neglect in breastfeeding becomes apparent in cases where mothers and newborns may require more specialized care and counselling. This includes mothers who have undergone caesarean delivery, infants admitted to the nursery, small and preterm babies who require expressed breastmilk, adolescent, first-time mothers, and HIV positive mothers.

#### *Neglect after caesarean section  *

Participants reported incidences where women who had delivered through a caesarean section would experience delays in initiating breastfeeding, particularly in cases where a mother had no guardian (a relation or friend) who would assist the mother to breastfeed the baby.

*“We had a case [where the mother had a] caesarean section and the baby was taken to the nursery but it took almost three hours because the patient had no guardian (caregiver) to take care of the baby . . . Caesarean section patients mostly rely on the guardians to take care of the babies in the nursery. After the mother was admitted to the postnatal ward, the staff in the postnatal ward forgot that the patient had no guardian and the baby was left unfed for hours. It was upon the inquiries by the nursery staff about the baby that it was found that there was more to the case.” Tertiary hospital nursing officer*

*Ok, so another situation, it’s when the mother has come to feed yet this mother has caesarean section on, now she is not able to maintain this holding position because of the pain on the wound . . . Ok, so these are some difficult situations that women encounter when holding babies.” District hospital clinical officer*

##### Neglect among small and sick newborns

Healthcare workers reported that other mothers were uncomfortable to handle small and sick newborns on their own which would delay initiation of breastfeeding and continuity of it. This lack of confidence would result in improper breastfeeding positions which would compromise the process as well as put the life of the newborn at risk.

*“ . . . some mothers are scared to hold a sick baby in their hands, ok? So, such mothers have poor holding, for example, I saw a certain mother is breastfeeding and she is leaning her back forward while keeping the baby flat, ok, this is a bad position as the baby can easily aspirate some milk . . . maybe she is scared to hold this baby upright, the baby is very sick, or maybe this baby is receiving some oxygen and then she is afraid to feed or hold the baby. District hospital clinical officer”*

*“Some will say me . . . I don’t want to breastfeed my child, because I am afraid the child may get HIV positive . . . Like the scenario that I once had . . . the mother was positive . . . she said she will not breastfeed the child but rather she will buy formula milk. She said . . . she already had two children [while she was HIV positive and] the children tested negative, so she was willing to go through that again . . . that she is using herbal medicine (instead of antiretroviral medication) and she doesn’t want the children to take even Nevirapine syrup and she was going to buy formula milk.” Tertiary hospital nurse*

*“We try to monitor … and ask if there are any concerns, like swollen or bleeding tits because that is a problem . . . It means the baby is at risk . . . of HIV and AIDS (for HIV positive mothers)” District hospital nurse*

#### Breastfeeding among adolescent and first time mothers

Adolescent, first-time mothers, attend less antenatal care, and have unique breastfeeding concerns that require additional counselling.

*“Do I need to mention the children? Those girls that are just giving birth at an early age, we have seen thirteen years, fourteen years, they still occur in our facilities. Those are unable even to breastfeed their baby! You have to find their mother or their grandmother. They are the ones who put the baby on the belly to breastfeed these children. So the challenges are there for those kids because they were not attending antenatal care so they don’t have enough information.” District hospital clinical officer*

*“Cosmetic, I mean, some of the ladies would say, “I don’t want to breastfeed because I don’t want my breast, you know, to fall.” They don’t want their breast to be loose. They want to be firm as if they haven’t given birth, so they would rather not breastfeed.” Tertiary hospital nurse*

### Revitalizing breastfeeding using the systems framework for health policy

1. Governance

Applied at the facility level, governance includes strengthening breastfeeding support through legislation of policies, organization of services, and quality assurance. Participants shared that health facilities could strengthen guidelines on roles and responsibilities for breastfeeding support instead of assuming that the mother received counselling with a previous health professional and address communication procedures between wards for continuity of care.

*“Uhh, like here in postnatal I would say, when the mothers are coming we assume that they were told in labor ward because some will come already breastfeeding but some not yet.” Tertiary hospital nurse*

To support breastfeeding guidelines, policies to ensure adequate staffing in maternity wards, and supportive supervision were highlighted by health workers.

*“I don’t think it’s (implementing breastfeeding policies) difficult or it’s complicated. It’s just that people will always need constant reminders . . . that constant reminder to be able to drive people to just initiate it.” District medical officer*

“*No that is a problem like I said there is understaffing in most facilities so we end up prioritizing the more needy mothers and babies, we may visit once after birth just to make sure baby has started breastfeeding but not a regular schedule as one may need to . . . Sometimes we ask for assistance from other wards, maybe a nurse can come in and help. If severe cases, we ask for someone who is home to come, but that is in rare circumstances because its likely to affect the next days staffing as well. NMT Mulanje”*

*I think the unit is quite big and the staffing levels do not match; we don’t have adequate members of staff to make sure they observe every feeding time. Sometimes when they is a need they will prioritize the babies that need to be monitored. Registrar QECH*

2. Knowledge

Within a systems approach to strengthening breastfeeding practices at health facilities, knowledge relates to understanding local situations, current gaps, and how best to disseminate and promote uptake. A cross-cutting theme across findings was a familiarity with breastfeeding as part of routine neonatal care but gaps are found when specialized care and counselling as needed. Health workers are trained about breastfeeding in pre-service curricula as well as receive periodic training to strengthen knowledge but shared that they wanted to learn more about practical skills on supporting mothers and procedures when encountering challenges, such as when mothers or newborns have health complications.

*“I think issues of those sick babies . . . because for the sick ones, we admit them in a nursery while mothers [are] in the postnatal ward, so there is a gap. So there is a need to learn how we can promote lactation between these separated baby and mom . . . ” Tertiary hospital nursing officer*

*“I think more of the practical skills; things like how to help women who are having problems of lactation, how to help them increase milk production, how to help them if they have sore breast, it’s more about how to support the mothers.” Pediatric specialist at a tertiary hospital*

There was also the need for better engagement with mothers. Health workers highlighted the need to evaluate the level of pre-existing knowledge learned during antenatal counselling, which may vary between different mothers. Health workers emphasized the need to teach by demonstration.

*“The most important thing is providing this support to women during lactation is when you are there, other than just explaining to them . . . What was happening was [that] we could just go in there and preach about it (breastfeeding) and then never demonstrate, never be there with them to support them, and even to supervise if they are doing it correctly or not . . . Most of our women here are, their literacy levels are very low, so if you just explain, most of the times, they don’t even understand and if you are explaining, even if they have questions, they are always scared they can’t ask. But when you are there and supporting them, it’s very helpful.” District hospital nurse*

3. Advocacy

Advocacy on breastfeeding includes community engagement and empowerment. At the health facility, this entails the inclusion of family members accompanying the mother. Older relatives in particular, such as grandmothers, hold significant household decision-making power. While they are there to support the mother, they may provide contradicting advice when they are not included in counselling.

*“She was old; she was a grand mum. So, when the granddaughter delivered, I was like the child should be breastfed, then the grandmother said no . . . so I would say we delayed to start the baby on the breast milk . . . At the end of the day, she agreed, and then the baby was fed.” Tertiary hospital nurse*

*“So small (young) mums are supported in our culture. Mostly, the mum will come with the grandmother, this is the mother who has vast experience in baby caring. If it’s not the grandmother, maybe the other mother who has also had some babies. We call it* Ntchembele *in our language. So a [young] mother or a new mother always comes with a senior or more experienced mothers so the first support that they get is from their immediate relatives who are these senior mothers . . . ” District clinical officer*

*“We advise them (new mother) how to feed the baby, then the [grand] mother will come in with a different story, giving her a different lesson on how to hold the baby . . . also telling her that the breast can block the baby’s nose!” Tertiary hospital nurse*

The inclusion of men in counselling is important to support their partners better. They expressed the need for more information. If they were also counselled, male partners expressed feeling included in the care of the mother and baby and were more willing to help.

*“I felt good, as one of the parents I am supposed to practice what they have told us, helping the mother doing it, and in the end, the mother may also appreciate that I am taking part because I was trained.” District hospital father*

4. Capacity- task shifting of roles

Within the context of staffing shortages within the health sector, health workers highlighted how task-shifting could strengthen the capacity to provide adequate breastfeeding counselling, follow-up, and care. In Malawi, there is a cadre of lay health workers at health facilities called patient attendants. Some participants highlighted that they may already be available and supporting mothers in the wards but need further specific training and formalization of their roles for breastfeeding support.

*“We have also what we call patients attendants who look after the babies and supervise the women when they are breastfeeding the babies.” Tertiary hospital nurse*

*“The first thing is the training of the health workers, these pieces of training should not only focus the trained health workers like clinician and nurses but if they can include ward attendants because these people may assist us in case the doctor or nurses are busy . . . ” District hospital clinical officer*

## Discussion

In explorations of both the quantitative and qualitative datasets, our study has shown that breastfeeding is a familiar but neglected practice. While early and exclusive breastfeeding practices among routine neonatal care scored high in the facility assessment, there were lower scores among those needing special care. Interviews likewise found that breastfeeding was accepted as a social norm among both mothers and health workers yet those requiring additional care such as women who delivered by caesarean section, infants with clinical complications, and women suffering from maternal morbidities were not adequately reached. Revitalizing breastfeeding support will need to be multi-sectoral and inclusive of all stakeholders. Strengthening facility-based breastfeeding support will require instituting supportive policies with adequate human resources and supervision, improving the knowledge base of health workers and mothers, empowering grandmothers, aunts and fathers who support the mother, and shifting some supportive roles to other non-medically trained health workers.

The normalization of breastfeeding as illustrated in the facility scores and qualitative findings is similar to earlier findings that described the conceptualization of breastfeeding as natural with the expectation that women adapt to it [17]. Another study found that health workers rarely promoted it because it was considered natural [10]. Our study contends that policies should highlight the need for continuous breastfeeding support with supportive supervision from management. This finding builds on other studies that have highlighted gaps in the available policies, such as emphasizing components of best practices but not others such as exclusive breastfeeding than early initiation in a Ghanaian study [18] or policies that are inconsistently applied [19–21]. Although previous ratings scored Malawi at 75% on implementation of policies on Infant and young child feeding in 2008, this rating needs to be reviewed against current trends and strengthened to ensure that activities in the facilities are implemented with fidelity [22]. Earlier studies have advocated for supervision by managers at a local facility to strengthen breastfeeding practices [11, 23], particularly to address the problems that result from inconsistent messaging and application of policies on breastfeeding [21]. Our study highlights a need for supportive supervision for initiation and follow-up, especially in cases with infants that are medically unstable to breastfeed or vulnerable populations such as adolescent mothers and HIV positive mothers. This was apparent in the facility assessments where scores were lower for neonates that needed special care and was further corroborated by the interviews.

Building on previous studies that recommend strengthening the knowledge base of health workers and mothers to support awareness of breastfeeding benefits throughout prenatal to postpartum periods [24–27], our study highlights gaps in knowledge on the management of women and neonates that have challenges to breastfeeding and explains the low scores in such neonates. These gaps have been reported earlier among primiparous women [28] teenage mothers [29] skills in expressing milk, management of breast engorgement, and painful and cracked nipples [30]. Updating the current breastfeeding training manuals with problem-based training for healthcare workers and mothers addressing the specific problematic issues would be a step towards strengthening breastfeeding practices. Additionally, continued engagement with health workers for knowledge sharing and discussion of practical skills and solutions to problems that arise from their experiences can build on the foundation in breastfeeding knowledge from pre-service training. We argue that knowledge would be optimized if it is tailor-made to fit the condition of a mother-baby pair and emphasized skill-building, for example, such as follow-up to recognize if a mother perceives insufficient breastmilk production and checking on the positioning of the infant to improve breastfeeding.

Although our study reported that healthcare workers are the main implementers and educators of breastfeeding, there is frequently a shortage of healthcare workers to provide care to all women adequately. Our study advocates for task-shifting of the roles of education and supporting mothers with breastfeeding and cement what has been reported recently in Malawi on using lay workers to strengthen breastfeeding support [31]. These supportive health workers could provide expert opinions to motivate and support women as they breastfeed [25] and close the gap in healthcare workers [26]. Interestingly, participants in our study shared that lay health workers may already be providing such support in their health facilities but their roles and responsibilities are not formalized. Auxiliary health workers may not also be trained, which challenges their ability to effectively support appropriate breastfeeding practices and recognize complications. For example, a South African study found that while a majority of nurses had positive attitudes and were strongly against the use of prelacteal feeds, nursing auxiliaries were largely negative towards the promotion of breastfeeding, and did not believe in or teach women about the benefits of breastfeeding [32]. A Nigerian study also found that while breastfeeding guidelines and policies were implemented, none of the auxiliary health workers were aware and had poor knowledge of early breastfeeding policies and benefits [33]. While task-shifting to lay health workers can reduce the burden on health professionals, training on breastfeeding practices and skills to mitigate challenges as well as supportive supervision is also required for lay health workers to adequately support women and their families.

Empowering a male partner and other members of the family to support a woman during breastfeeding is a concept embedded with family-centered care. This has been advocated previously by other researchers. An earlier Malawian study showed that men had low levels of knowledge on breastfeeding despite being influential household decision-makers [34] and a Ghanaian study found that partner supporter was an enabler to breastfeeding [29]. Our findings of grandmothers and aunts sometimes being a barrier to breastfeeding resonates with a Ghanaian study that reported mothers not being allowed to breastfeed especially at night because influential relatives, such as grandmothers, would not allow them [35]. In addition to postpartum counselling at the health facility, family members may be empowered through community sensitization that targets problematic areas with breastfeeding and early initiation [18, 29] and this could be done through mass media [36]. There is a need for more research on the role of mass media to promote optimal breastfeeding practices and to advocate for husbands and partners, relatives, and other community members to support breastfeeding mothers. To improve exclusive breastfeeding support in health facilities in Malawi we provide a summary of recommendations as illustrated in Table 2.

**Table 2:** Recommendations to improve exclusive breastfeeding support in health facilities in Malawi

● Strengthening the knowledge base of both pregnant and postpartum women	
● Development of problem based learning materials for healthcare workers to facilitate breastfeeding support.	
● Engagement and empowerment of other family members on breastfeeding practices and their role in supporting a breastfeeding woman.	
● Task-shifting the implementation of breastfeeding support to trained lay healthcare workers to avoid overwhelming the nursing staff.	
● Strengthening implementation of policies on breastfeeding and ensuring adequate funding to implement outlined activities.	

## Conclusions

Our study triangulated facility assessment findings with perspectives of health workers and mothers, grandmothers, aunts and fathers at Malawian hospitals and consistently found that breastfeeding was normalized as routine, which led to gaps in supporting the practice particularly for vulnerable populations. Neglect in breastfeeding support among vulnerable populations exacerbates health inequities and may lend to perpetuating social inequalities through the long-term effects of breastfeeding on human capital. Health systems can be strengthened through family-centred hospital care to recognize and support women experiencing breastfeeding challenges to ensure their needs are met.

## References

[1] Darmstadt GL, Bhutta ZA, Cousens S, Adam T, Walker N, de Bernis L (2005). Evidence-based, cost-effective interventions: how many newborn babies can we save?. Lancet..

[2] Jones G, Steketee RW, Black RE, Bhutta ZA, Morris SS (2003). Bellagio child survival study group. How many child deaths can we prevent this year?. Lancet..

[3] Victora CG, Bahl R, Barros AJD, França GVA, Horton S, Krasevec J, Murch S, Sankar MJ, Walker N, Rollins NC (2016). Breastfeeding in the 21st century: epidemiology, mechanisms, and lifelong effect. Lancet..

[4] Victora CG, Horta BL, De Mola CL, Quevedo L, Pinheiro RT, Gigante DP, Gonçalves H, Barros FC (2015). Association between breastfeeding and intelligence, educational attainment, and income at 30 years of age: a prospective birth cohort study from Brazil. Lancet Global Health.

[5] Rollins NC, Bhandari N, Hajeebhoy N, Horton S, Lutter CK, Martines JC, Piwoz EG, Richter LM, Victora CG (2016). Why invest, and what it will take to improve breastfeeding practices?. Lancet..

[6] Bhattacharjee NV, Schaeffer LE, Marczak LB, Ross JM, Swartz SJ, Albright J, Gardner WM, Shields C, Sligar A, Schipp MF, Pickering BV, Henry NJ, Johnson KB, Louie C, Cork MA, Steuben KM, Lazzar-Atwood A, Lu D, Kinyoki DK, Osgood-Zimmerman A, Earl L, Mosser JF, Deshpande A, Burstein R, Woyczynski LP, Wilson KF, VanderHeide JD, Wiens KE, Reiner RC, Piwoz EG, Rawat R, Sartorius B, Davis Weaver N, Nixon MR, Smith DL, Kassebaum NJ, Gakidou E, Lim SS, Mokdad AH, Murray CJL, Dwyer-Lindgren L, Hay SI (2019). Mapping exclusive breastfeeding in Africa between 2000 and 2017. Nat Med.

[7] Gupta A, Dadhich JP, Rundall P, Bidla N (2019). Interpreting the world health assembly targets on exclusive breastfeeding by 2025: what is expected of each country?. World Nutr.

[8] Doherty T, Horwood C, Haskins L, Magasana V, Goga A, Feucht U, Sanders D, Tylleskar T, Kauchali S, Dhansay MA, Rollins N, Kroon M, Engebretsen IMS (2020). Breastfeeding advice for reality: Women’s perspectives on primary care support in South Africa. Matern Child Nutr.

[9] Senbanjo IO, Oshikoya KA, Ogbera OA, Wright KO, Anga AL (2014). Breastfeeding policy and practices at the general paediatric outpatient clinic of a teaching hospital in Lagos, Nigeria. Int Breastfeed J.

[10] Moussa Abba A, De Koninck M, Hamelin AM (2010). A qualitative study of the promotion of exclusive breastfeeding by health professionals in Niamey, Niger. Int Breastfeed J.

[11] Agbozo F, Ocansey D, Atitto P, Jahn A (2020). Compliance of a baby-friendly designated hospital in Ghana with the WHO/UNICEF baby and mother-friendly care practices. J Hum Lact.

[12] Fetters MD, Curry LA, Creswell JW (2013). Achieving integration in mixed methods designs - principles and practices. Health Serv Res.

[13] Nyondo-Mipando AL, Kinshella M-LW, Salimu S, Chiwaya B, Chikoti F, Chirambo L, Mwaungulu E, Banda M, Newberry L, Njirammadzi J, Hiwa T, Vidler M, Dube Q, Molyneux E, Mfutso-Bengo J, Goldfarb DM, Kawaza K (2020). “It brought hope and peace in my heart:” caregivers perceptions on kangaroo mother care services in Malawi. BMC Pediatr.

[14] Kawaza K, Kinshella MLW, Hiwa T, Njirammadzi J, Banda M, Vidler M, Newberry L, Nyondo-Mipando AL, Dube Q, Molyneux E, Goldfarb DM (2020). Assessing quality of newborn care at district facilities in Malawi. BMC Health Serv Res.

[15] Braun V, Clarke V (2006). Using thematic analysis in psychology. Qual Res Psychol.

[16] Commonwealth Secretariat. A Systems Framework for Healthy Policy advancing, global health security and sustainable well-being for all.The Commonwealth Secretariat (2016). https://www.thecommonwealth-healthhub.net/systems-framework-healthy-policy/. (last Accessed on 11 August 2021)

[17] Hasselberg M, Huus K, Golsäter M (2016). Breastfeeding preterm infants at a neonatal care unit in rural Tanzania. J Obstet Gynecol Neonatal Nurs.

[18] Tawiah-Agyemang C, Kirkwood BR, Edmond K, Bazzano A, Hill Z (2008). Early initiation of breast-feeding in Ghana: barriers and facilitators. J Perinatol.

[19] Nii Okai Aryeetey R, Antwi CL (2013). Re-assessment of selected baby-friendly maternity facilities in Accra, Ghana. Int Breastfeed J.

[20] Nabwera HM, Jepkosgei J, Muraya KW, Hassan AS, Molyneux CS, Ali R, Prentice AM, Berkley JA, Mwangome MK (2017). What influences feeding decisions for HIV-exposed infants in rural Kenya?. Int Breastfeed J.

[21] Morgan JJ, Jeggels JD (2015). Factors influencing the infant feeding choices of HIV-positive mothers at a level two hospital in Cape Town. Afr J Midwifery Womens Health.

[22] Malawi Ministry of Health Nutrition Unit. The World Breastfeeding Trends Initiative: Malawi Assessment Report (2008). https://www.worldbreastfeedingtrends.org/wbti-country-report.php. (Last Accessed on 11 August 2021).

[23] Kalisa R, Malande O, Nankunda J, Tumwine JK (2015). Magnitude and factors associated with delayed initiation of breastfeeding among mothers who deliver in Mulago hospital, Uganda. Afr Health Sci.

[24] Chipojola R, Chiu HY, Huda MH, Lin YM, Kuo SY (2020). Effectiveness of theory-based educational interventions on breastfeeding self-efficacy and exclusive breastfeeding: a systematic review and meta-analysis. Int J Nurs Stud.

[25] Acheampong AK, Aziato L, Marfo M, Amevor P (2020). Breastfeeding and caring for children: a qualitative exploration of the experiences of mothers with physical impairments in Ghana. BMC Pregnancy Childbirth.

[26] Shobo OG, Umar N, Gana A, Longtoe P, Idogho O, Anyanti J (2020). Factors influencing the early initiation of breast feeding in public primary healthcare facilities in Northeast Nigeria: a mixed-method study. BMJ Open.

[27] Gejo NG, Weldearegay HG, Wtinsaie KT, Mekango DE, Woldemichael ES, Buda AS (2019). Exclusive breastfeeding and associated factors among HIV positive mothers in northern Ethiopia. PLoS One.

[28] Minas AG, Ganga-Limando M (2016). Social-cognitive predictors of exclusive breastfeeding among primiparous mothers in Addis Ababa, Ethiopia. PLoS One.

[29] Acheampong AK, Ganga-Limando M, Aziato L (2020). Perceived enablers of exclusive breastfeeding by teenage mothers in Ghana. S Afr Fam Pract.

[30] Daniels L, Jackson D (2011). Knowledge, attitudes and practices of nursing staff regarding the baby-friendly hospital initiative in non-accredited obstetric units in Cape Town. South African J Clin Nutr.

[31] Kavle JA, Welch PR, Bwanali F, Nyambo K, Guta J, Mapongo N, Straubinger S, Kambale S (2019). The revitalization and scale-up of the baby-friendly hospital initiative in Malawi. Matern Child Nutr..

[32] Mgolozeli SE, Shilubane HN, Khoza LB (2019). Nurses’ attitudes towards the implementation of the mother-baby friendly initiative in selected primary healthcare facilities at Makhuduthamaga municipality, Limpopo province. Curationis.

[33] Okolo SN, Ogbonna C (2002). Knowledge, attitude and practice of health workers in Keffi local government hospitals regarding baby-friendly hospital initiative (BFHI) practices. Eur J Clin Nutr.

[34] Chintalapudi N, Hamela G, Mofolo I, Maman S, Hosseinipour MC, Hoffman IF, Flax VL (2018). Infant and young child feeding decision making and practices: Malawian mothers’ and fathers’ roles in the context of HIV. J Hum Lact.

[35] Mensah KA, Acheampong E, Anokye FO, Okyere P, Appiah-Brempong E, Adjei RO (2017). Factors influencing the practice of exclusive breastfeeding among nursing mothers in a peri-urban district of Ghana. BMC Res Notes.

[36] Wainaina CW, Wanjohi M, Wekesah F, Woolhead G, Kimani-Murage E (2018). Exploring the experiences of middle income mothers in practicing exclusive breastfeeding in Nairobi, Kenya. Matern Child Health J.

